# Wide Incidence Angle-Insensitive Metamaterial Absorber for Both TE and TM Polarization using Eight-Circular-Sector

**DOI:** 10.1038/s41598-017-03591-2

**Published:** 2017-06-09

**Authors:** Toan Trung Nguyen, Sungjoon Lim

**Affiliations:** School of Electrical and Electronics Engineering, Chung-Ang University, Heukseok-Dong, Dongjak-Gu 156-756 Republic of Korea

## Abstract

In this paper, a wide incidence angle-insensitive metamaterial absorber is proposed using eight-circular-sector (ECS). Under normal incidence, the proposed absorber shows high absorptivity at different polarizations due to its symmetric geometry. Under oblique incidence, zero-reflection conditions for transverse electric (TE) and transverse magnetic (TM) polarization are different. Nevertheless, the proposed absorber shows high absorptivity under oblique incidence of both TE and TM polarization due to ECS. The performance of the proposed absorber was demonstrated with full-wave simulation and measurements. The simulated absorptivity at the specular angles exceed 90% and the frequency variation is less than 0.7% at approximately 9.26 GHz up to a 70° incidence angle in both TM and TE polarization. We built the proposed absorber on a printed-circuit board with 20 × 20 unit cells, and we demonstrated its performance experimentally in free space. The measured absorptivity at 9.26 GHz for the specular angles is close to 98% for all polarization angles under normal incidence. As the incidence angle is varied from 0° to 70°, the measured absorptivity at 9.26 GHz for the specular angles remain above 92% in both TE and TM polarization.

## Introduction

Electromagnetic (EM) metamaterials (MMs) are artificial materials engineered to exhibit unique properties not found in nature^1^—for example, in terms of tailoring their effective permittivity or permeability. Metamaterials can be realised using a periodic array of resonators, such as the split-ring resonator (SRR)^2^. Because of their extraordinary features, they have been used for many applications, such as compact microwave circuits^3^, super lenses^4^, miniaturised wireless components^5^, and absorbers^6^ in the microwave and terahertz bands.

In particular, the MM absorber has generated particular interest since it was introduced by Landy in 2008^7^. An MM absorber generates electric and magnetic resonances that are controlled independently to tailor the effective permittivity and permeability, respectively. MM absorbers have some advantages compared with conventional absorbers such as ferrite, wedge-tapered absorbers^8^, and Salisbury screen absorbers^9^. For instance, MM absorbers can achieve a high level of absorptivity even with a thin substrate. In addition, functional absorbers can be realized with tunable devices or materials. These advantages of MM absorbers have motivated research into various applications in the microwave through the optical regime, such as photodetector^10–17^, solar photovoltaic^18, 19^, and thermo-photovoltaic^20, 21^ applications. Because an MM absorber involves a periodic array of resonators, it operates at a specific frequency and has a narrow bandwidth. Some studies on broadband MM absorbers have sought to increase the bandwidth^22–24^.

In general, the absorptivity of an MM absorber depends also on the incident polarization and angle. Polarization-independent MM absorbers can be easily achieved by designing a horizontally and vertically symmetric unit cell^25, 26^. On the other hand, it is difficult to design incidence angle-independent MM absorbers. Therefore, many researchers have proposed incidence angle-insensitive MM unit cells such as the circular sector^27^, split-ring cross resonator (SRCR)^28^ and surrounding via array^29^, subwavelength unit cell in a multi-layer^30^, and four-fold rotational symmetric electric resonator with a cross-printed bottom^31^. However, previous angle-insensitive MM absorbers showed high absorptivity under wide incidence angles with only transverse magnetic (TM) polarization. Their absorptivity decrease and absorption frequencies are changed under wide incidence angles with transverse electric (TE) polarization^26–33^.

Therefore, in this paper, we propose a novel wide incidence angle-insensitive MM absorber for both TE and TM polarization. In order to achieve high absorptivity under oblique incidence with both TE and TM polarization, an eight-circular-sector (ECS) is introduced as the unit cell of the proposed MM absorber. The vertically and horizontally symmetric geometry of the proposed unit cell gives rise to polarization insensitivity. The performance of the proposed absorber is demonstrated under normal and oblique incidences with a full-wave simulation and measurements. In addition, the absorptivity and frequency variation of the proposed absorber are compared with those of the previous wide angle-insensitive MM absorbers.

## Principle of the metamaterial absorber

An EM wave incident onto an MM surface is reflected and transmitted. High absorptivity can be achieved by minimizing both the reflection coefficient Γ(ω) and the transmission coefficient T(ω), because the absorptivity A(ω) is calculated as^19^

1 $$A(\omega )\,=\,1\,-\,\Gamma (\omega )\,-\,T(\omega )$$

Under normal incidence, the reflection coefficient is given by

2 $$\Gamma (\omega )\,=\,\frac{Z(\omega )\,-\,{Z}_{0}}{Z(\omega )\,+\,{Z}_{0}},$$

where Z(ω) and Z_0_ are the impedances of the MM absorber and free space, respectively^19^. Therefore, the zero-reflection condition is satisfied when Z(ω) = Z_0_. Within the effective medium approximation, the MM is characterised by its frequency-dependent relative permeability (*μ*_*r*_) and permittivity (*ε*_*r*_). Its impedance can also be expressed as^19^

3 $$Z(\omega )\,=\,\sqrt{\frac{{\mu }_{0}{\mu }_{r}(\omega )}{{\varepsilon }_{0}{\varepsilon }_{r}(\omega )}},$$

4 $${Z}_{0}\,=\,\sqrt{\frac{{\mu }_{0}}{{\varepsilon }_{0}}}\,=\,377\,\Omega ,$$

where *ε*_0_ and *μ*_0_ are the permittivity and permeability, respectively, of free space. When Z(ω) is the same as Z_0_, the reflection coefficient becomes zero from Equation (2). Therefore, we can achieve a reflection coefficient of zero by tailoring *ε*_*r*_ and *μ*_*r*_ to be identical to each other. When there is no reflected wave, EM energy is transmitted and absorbed. When the bottom plane is fully conducted and the transmitted EM wave is dissipated from dielectric losses, we can achieve zero transmission^27^. Therefore, lossy dielectric materials are preferred for MM absorbers.

Meanwhile, as the incident angle increases, such as oblique incidence, the absorptivity decreases because of the reflected EM wave. This is unavoidable because the zero-reflection condition differs under normal and oblique incidences. For instance, at oblique incidence, the reflection coefficients for the perpendicular and parallel polarizations are given by^20^

5 $${\Gamma }_{\perp }(\omega )\,=\,\frac{Z(\omega )\cos \,{\theta }_{i}\,-\,{Z}_{0}\,\cos \,{\theta }_{t}}{Z(\omega )\cos \,{\theta }_{i}\,+\,{Z}_{0}\,\cos \,{\theta }_{t}},$$

6 $${\Gamma }_{||}(\omega )\,=\,\frac{Z(\omega )\,\cos \,{\theta }_{t}\,-\,{Z}_{0}\,\cos \,{\theta }_{i}}{Z(\omega )\,\cos \,{\theta }_{t}\,+\,{Z}_{0}\,\cos \,{\theta }_{i}},$$

where *θ*_*i*_ and *θ*_*t*_ are the incident and transmission angles, respectively. Although the MM absorber shows perfect absorptivity under normal incidence, its absorptivity changes when the incident angles are varied as per Equations (2),(5) and (6). Therefore, an angle-insensitive unit cell must be designed for obtaining an incidence angle-insensitive MM absorber. In addition, the transmission coefficient can be minimized by increasing the loss factor. The refractive index (n) of an MM has a particularly large imaginary part, which corresponds to the loss factor. A transmitted wave is dissipated by a large loss factor in a substrate.

### Unit cell design

**Figure 1:**
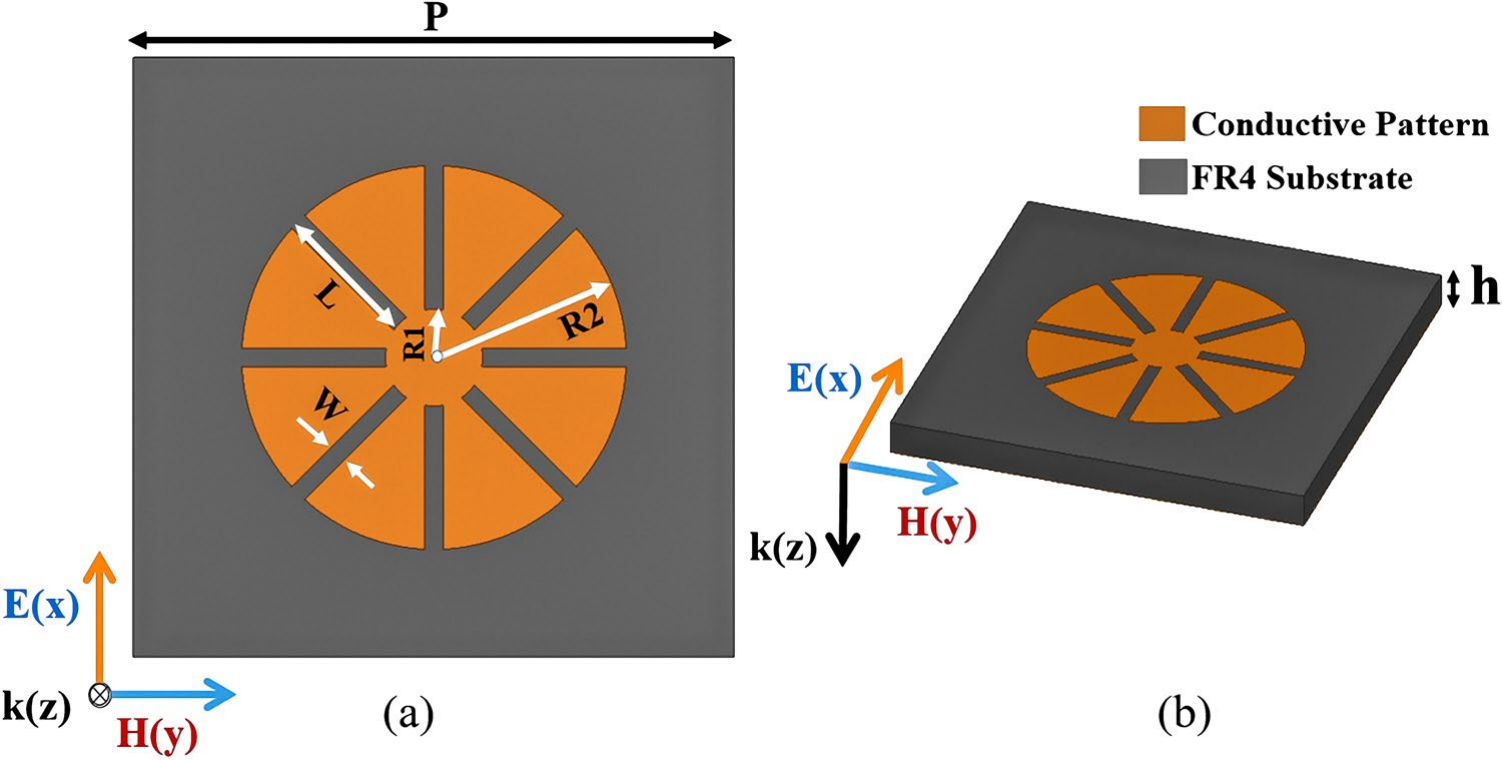
(**a**) Top view and (**b**) 3D view of a unit cell: P = 10 mm, R_1_ = 0.81 mm, W = 0.3 mm, h = 0.8 mm, R_2_ = 3.2 mm, L = 2.4 mm.

Figure 1 shows the top and 3D view of the proposed unit cell. The top of the unit cell consists of the eight-circular-sector (ECS), and the bottom layer is fully covered with a copper sheet. As shown in Fig. 1, the ECS design is characterized by four parameters (P, W, R_1_, and R_2_). P and W are the length of one side of the unit cell and the width of gap between each circular sector, respectively. R_1_ is the radius of the circular patch at the centre. R_2_ is the radius of a circular sector (R_2_ ≈ L + R_1_), where L is the length of the protruded circular sector. Because the unit cell is horizontally and vertically symmetric, its absorptivity is expected to be identical for all polarization angles (*ϕ*).

In this work, we used an FR-4 substrate with thickness h of 0.8 mm. Its relative permittivity and dielectric loss tangent are 3.9 and 0.02, respectively. In general, FR-4 substrates are not used for the X-band because of their high dielectric loss. However, FR-4 substrates are good candidates for absorber applications because of their high dielectric loss and low cost.

The ECS unit cell is designed on the basis of LC resonance^28^, and its resonant frequency is calculated by

7 $$f\,=\,\frac{1}{2\pi \sqrt{{L}_{eff}{C}_{eff}}}$$

**Figure 2:**
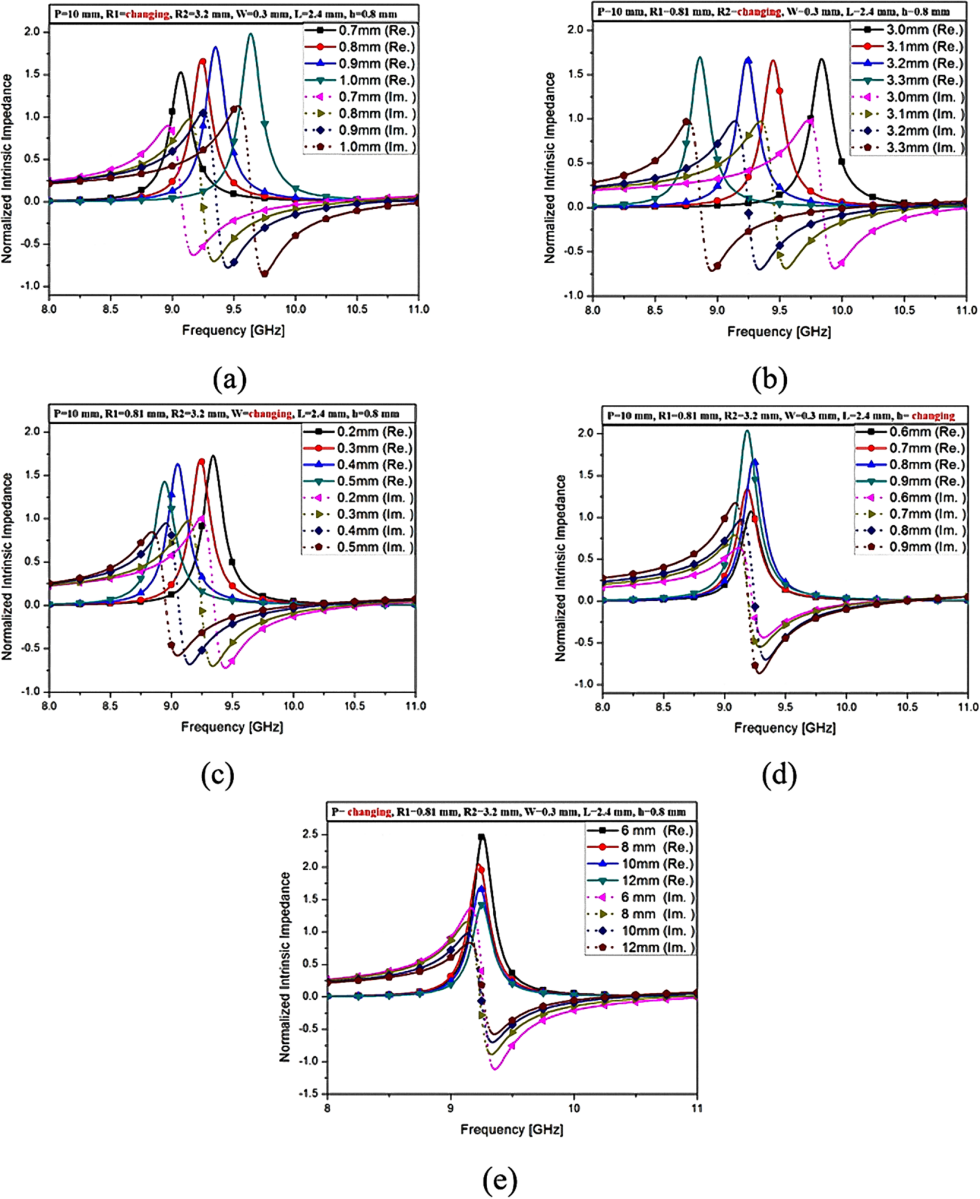
Simulated normalized complex impedances of the proposed MM absorber at different values of the parameters: (**a**) R_1_ varying from 0.7 mm–1.0 mm, (**b**) R_2_ varying from 3.0 mm–3.3 mm, (**c**) W varying from 0.2 mm–0.5 mm, (**d**) h varying from 0.6 mm–0.9 mm, (**e**) P varying from 6 mm–12 mm.

The effective inductance (*L*_*eff*_) is mainly determined from the length and width of the conductive pattern, such as R_1_, R_2_, and L. The effective capacitance (*C*_*eff*_) is mainly determined by the gap and length between each circular sector, such as L and W. Figure 2 shows the impedances of the proposed MM absorber when the geometrical parameters of the ECS are varied. Figure 2a shows that when R_1_ increases from 0.7 mm to 1.0 mm, the resonant frequency increases and the peak impedance is increased. However, when the circular patch size at the centre (R_1_) is larger given a slightly increased inductance, the gap’s length (L) becomes narrower because the other parameters (P, R_2_, W, h) are fixed. Therefore, the resonant frequency increases with larger R_1_ because a higher effective capacitance is decreased with larger R_1_ (smaller L). When R_2_ increases from 3.0 mm to 3.3 mm, the resonant frequency decreases and the peak impedance remains unchanged, as shown in Fig. 2b. The reason is that when R_2_ is larger, the inductance and capacitance are increased because the gap’s length (L) becomes larger, because the other parameters (P, R_1_, W, h) are fixed. Therefore, the resonant frequency decreases because of both higher inductance and capacitance with larger R_2_ (larger L). When W increases from 0.2 mm to 0.5 mm, the resonant frequency decreases and the peak impedance slightly decreases, as shown in Fig. 2c. The reason is that when W is larger, the other parameters (P, R_1_, R_2_, h) are fixed. Because of that, the resonant frequency decreases with larger W because the effective capacitance (*C*_*eff*_) is increased.

The thickness (h) and the length of one side (P) of the substrate both affect the peak impedance and slightly shift the resonant frequency. It is observed from Fig. 2d that when the thickness (h) is increased from 0.6 mm to 1.0 mm, the resonant frequency is not changed, and peak impedance is increased. As shown in Fig. 2e, when the length one side of the unit cell (P) is increased from 6 mm to 12 mm, the resonant frequency is not changed, and peak impedance is decreased. Finally, its parameters are determined as: P = 10 mm, R_1_ = 0.81 mm, W = 0.3 mm, h = 0.8 mm, R_2_ = 3.2 mm, and L = 2.4 mm.

**Figure 3:**
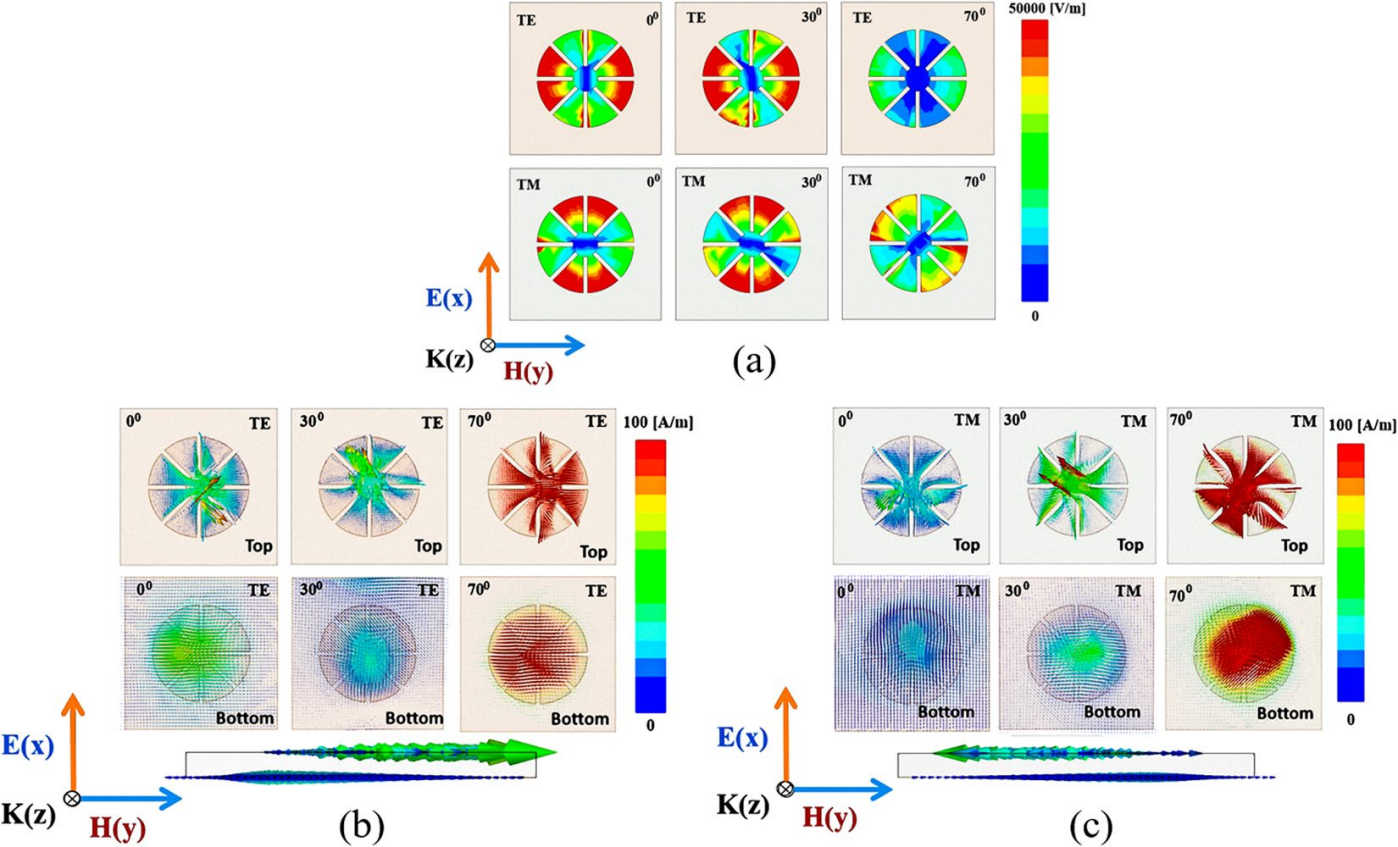
(**a**) Magnitude of electric-field distribution of the ECS unit cell: TE and TM modes under normal incidence (θ = 0°) and under oblique incidence (θ = 30° and θ = 70°). Simulated vector-current distribution of the top, bottom, and side of the ECS unit cell: (**b**) TE mode, (**c**) TM mode under normal incidence (θ = 0°) and under oblique incidence (θ = 30° and θ = 70°).

The fundamental principles of the MM absorber can be understood in terms of electric and magnetic resonances, visualized by plotting the electric-field magnitude and vector-current distributions^29–32^. Figure 3a shows the simulated magnitude of the electric field distributions of the ECS unit cell under normal incidence (*θ* = 0°) and under oblique incidence (*θ* = 30° and 70°) for both TE and TM modes. It is observed that the electric field is strongly coupled to the ECS. In addition, the antiparallel currents at the top and bottom of the unit cell under normal incidence (*θ* = 0°) and under oblique incidence (*θ* = 30° and 70°) for both TE and TM modes are shown in Fig. 3b and c, respectively. The circulating and antiparallel currents are responsive to the magnetic resonance. The antiparallel currents form a magnetic dipole that functions as a circulating current. The magnetic dipole direction is along the incident magnetic field polarization. Therefore, it strongly traps the incident magnetic energy, thus resulting in minimum reflection and strong absorption within a lossy dielectric material.

**Figure 4:**
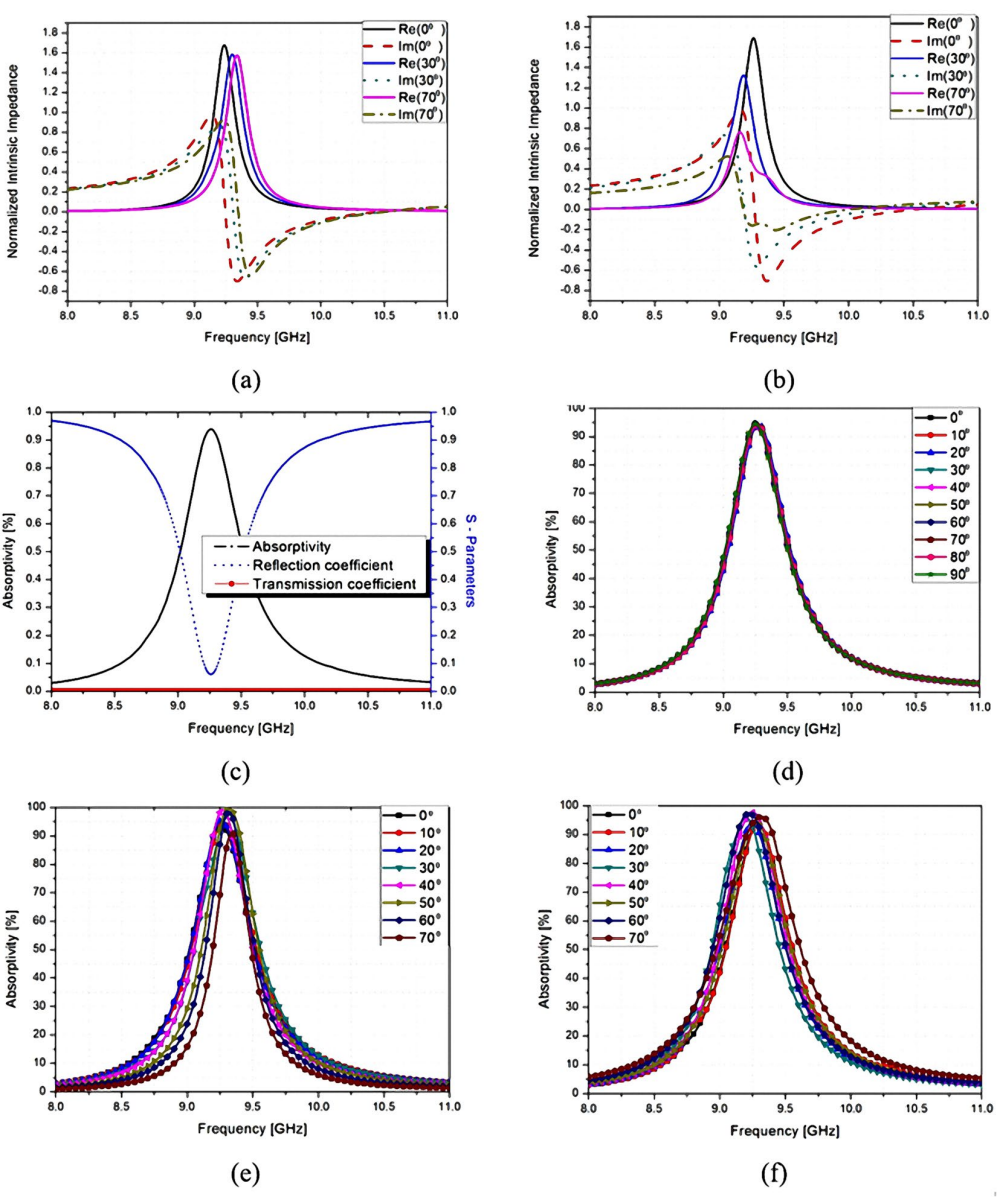
Simulated normalized impedances of the proposed MM absorber for different incident angles for (**a**) TE mode and (**b**) TM mode. (**c**) Simulated reflection coefficient, transmission coefficient, and absorptivity of the proposed MM absorber under normal incidence. (**d**) Simulated absorptivity of the proposed absorber for polarization angles ϕ ranging from 0° to 90° (**d**). Simulated absorptivity of the proposed MM absorber for incident angles θ ranging from 0° to 70° for (**e**) TE mode and (**f**) TM mode.

Figure 4a and b show the simulated complex impedance of the proposed MM, which is normalized with the impedance of free space in TE and TM modes for different incident angles (*θ*). Under normal incidence (*θ* = 0°) and oblique incidence (*θ* = 30° and *θ* = 70°), both TE and TM modes show the resonant frequency slightly changed at approximately 9.26 GHz, and the real part of the impedance is close to unity when the imaginary parts are zero. Therefore, it is expected that the proposed absorber operates at 9.26 GHz. From Fig. 4d, the simulated absorptivity at the specular angles is 96% at 9.26 GHz for all polarization angles (*ϕ*) ranging from 0° to 90°. Figure 4e and f show that the resonant frequency does not change until *θ* is 70°, and the absorptivity at the specular angles is maintained higher than 90% until 70° for the TM and TE modes, respectively.

### Fabrication and measurement results

**Figure 5:**
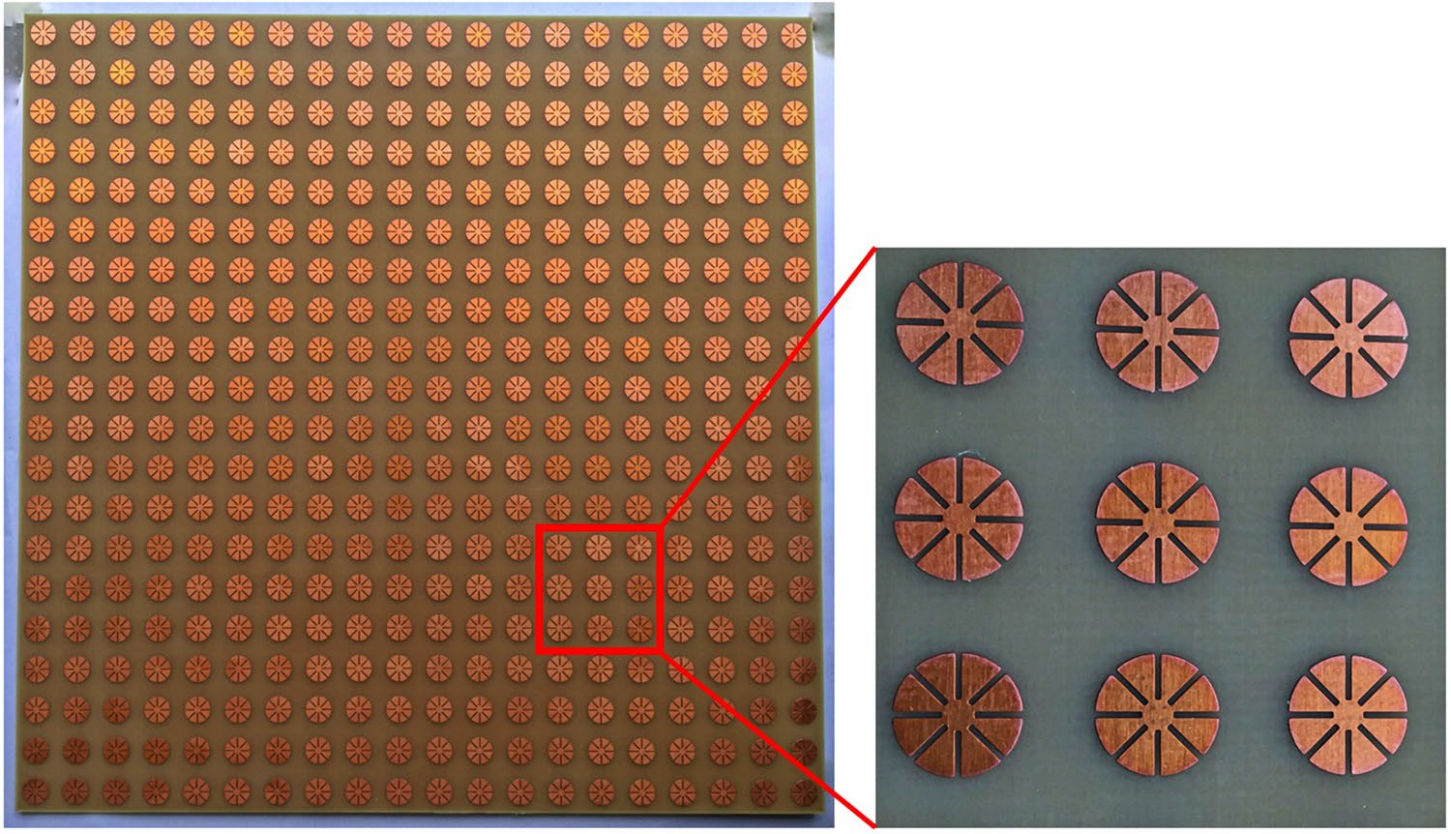
Fabricated metamaterial absorber prototype.

**Figure 6:**
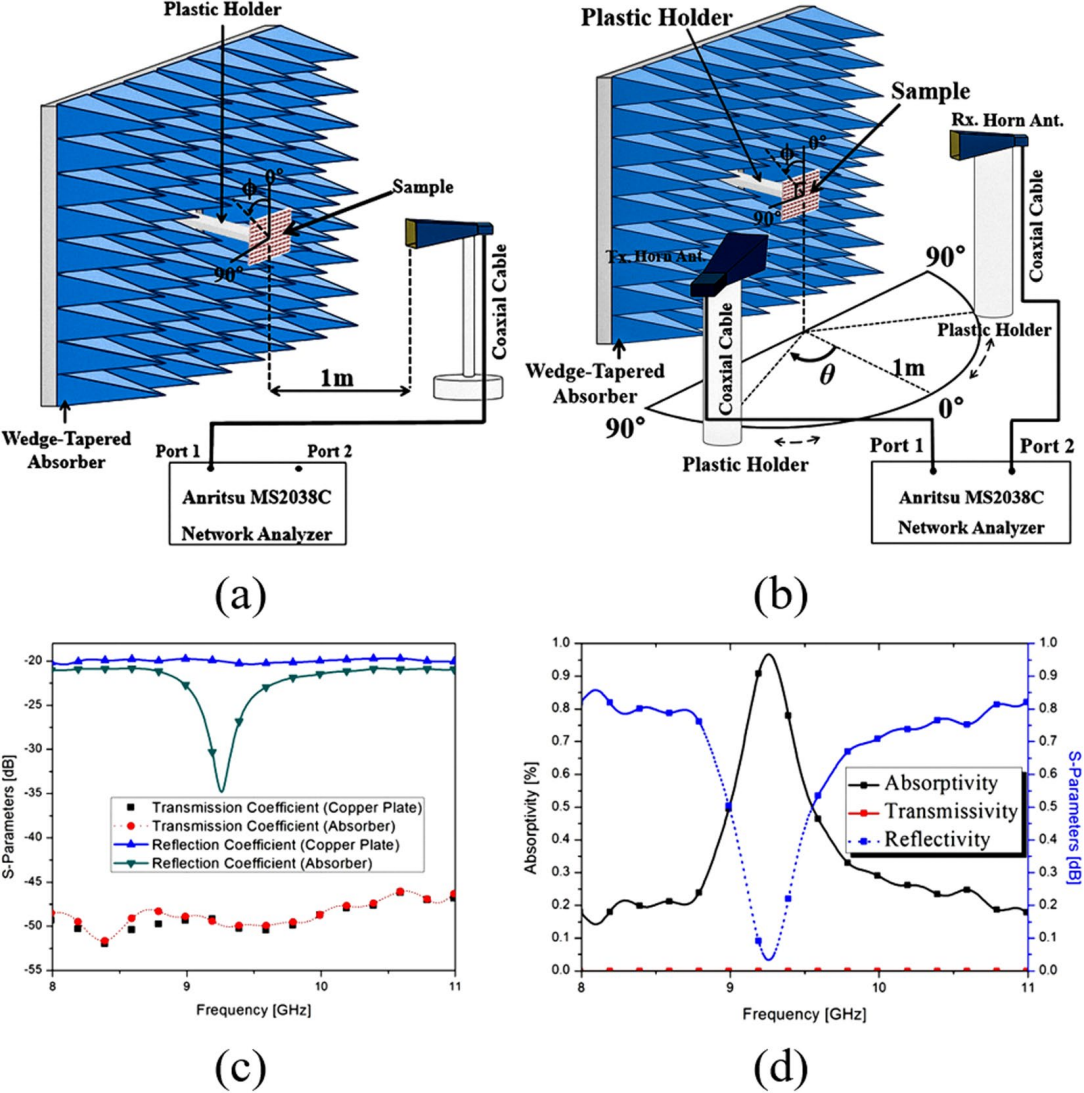
Free-space measurement setup for (**a**) normal incidence and (**b**) oblique incidence. (**c**) Measured S-Parameters of the absorber and a copper plate under normal incidence before calibration. (**d**) Measured reflectivity, transmissivity, and absorptivity of the proposed MM absorber under normal incidence after calibration.

In order to demonstrate the performance of the proposed MM absorber, a prototype with 20 × 20 unit cells was fabricated on an FR-4 substrate. Figure 5 shows a photograph of the fabricated prototype with an overall size of 200 mm × 200 mm. We used copper for the conductive patterns on the top and bottom planes. We measured the absorptivity of the fabricated prototype in free space. Figure 6a and b illustrates the measurement setup. We used an Anritsu MS2038C vector network analyzer (VNA) to measure the S-parameters. We used a single horn antenna to measure the S-parameters under normal incidence. The reflection coefficient and transmission coefficient are calculated from the S-parameters after calibration. Before measuring the S-parameters of the absorber prototype, we first measured the S-parameter of the copper plate and set the magnitude of its reflection coefficient to be 1 for the calibration process. Figure 6c shows the measured S-parameters of the copper plate and fabricated absorber before calibration. The transmission coefficient is almost zero because of the completely conductive bottom plane. From Equation (1), we calculated the absorptivity from the reflection coefficient and transmission coefficient. Figure 6d shows the measured reflectivity, transmissivity, and absorptivity at the specular angle of the proposed absorber after calibration. We observed that the measured absorptivity at the specular angle was 98% at 9.26 GHz, whereas the simulated absorptivity at the specular angle was 96% at 9.26 GHz. Therefore, the simulated and measured results showed the same resonant frequency.

**Figure 7:**
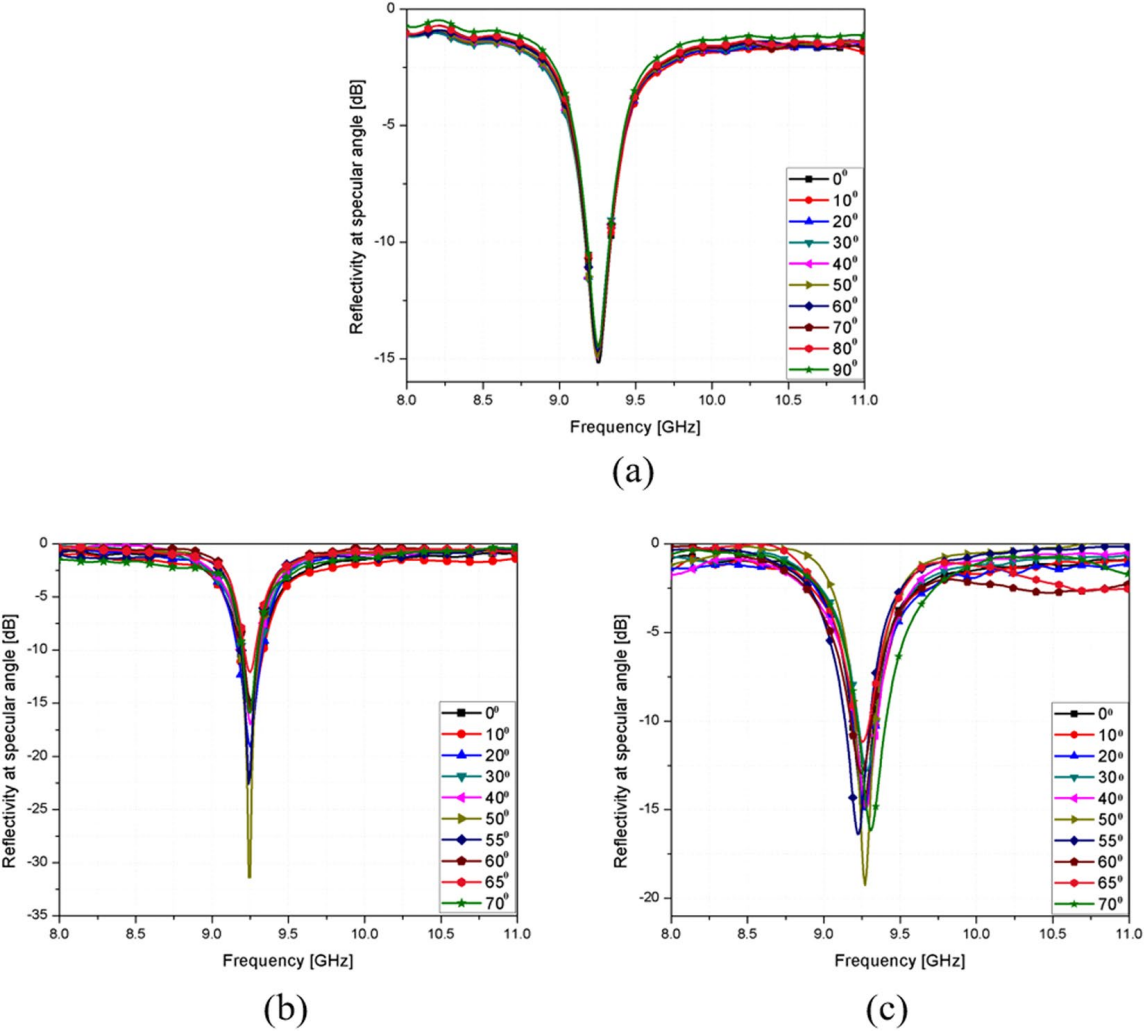
(**a**) Measured reflectivity of the proposed absorber for different polarization angles ϕ under normal incidence ranging from 0° to 90°. Measured reflectivity at the specular angle of the proposed absorber for incident angles θ ranging from 0° to 70° in (**b**) TE and (c) TM polarization.

In order to see the polarization sensitivity of the proposed absorber, the horn antenna was rotated for a polarization angle (*ϕ*) ranging from 0°–90° under normal incidence. Figure 7a shows the measured reflectivity at the specular angle of the proposed absorber for different polarization angles ϕ under normal incidence ranging from 0° to 90°. It was successfully demonstrated that the reflectivity and frequency of the proposed absorber were not changed for all polarization angles.

We used two horn antennas to measure the reflectivity at the specular angle under oblique incidence. One antenna was used to transmit EM energy, and its incident angle was changed by rotating the horn antenna from 0° to 70°. The other horn antenna was used to receive the EM energy reflected from the absorber, and it was placed at an angle to satisfy Snell’s law. Figure 7b and c show the measured reflectivity at the specular angle of the fabricated prototype at the TE and TM polarization, respectively. For the TM polarization, the reflectivity and resonant frequency remain unchanged up to *θ* = 70°, while maintaining a reflectivity of almost −12.5 dB at 9.26 GHz for the specular angles, and the peak reflection frequency varies from 9.24 GHz to 9.29 GHz. For the TE polarization, the reflectivity increases as the incident angle increases. However, a reflectivity lower than −11.5 dB is maintained up to *θ* = 70° at 9.26 GHz for the specular angles. Finally, lower than −11.5 dB reflectivity at 9.26 GHz for the specular angle is achieved up to *θ* = 70° for both TE and TM polarization. Therefore, we successfully demonstrated that the absorptivity for the specular angle of the proposed metamaterial absorber is insensitive to the incident angle of both TE and TM polarization.

**Figure. 8:**
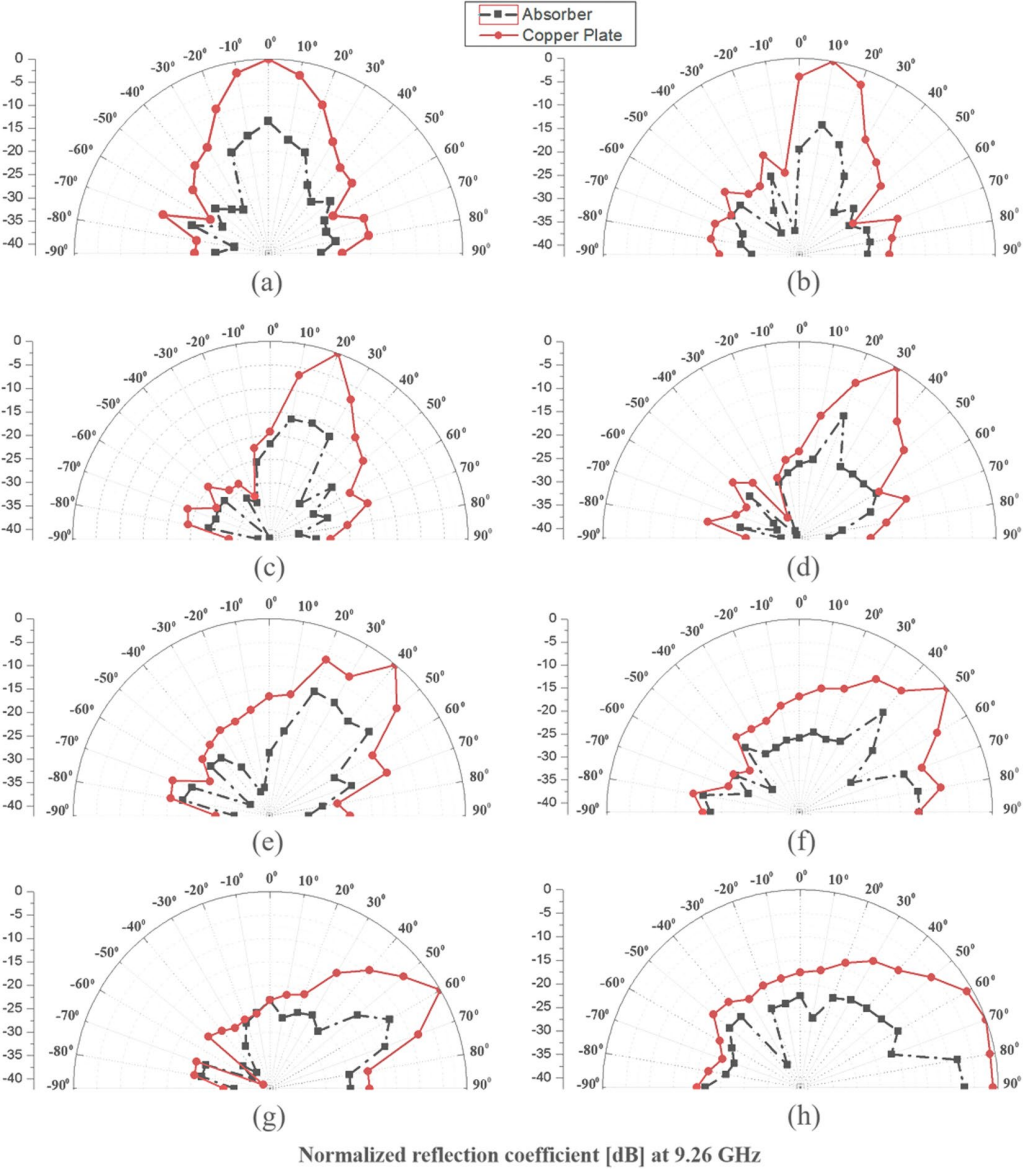
Bistatic RCS measurement results at 9.26 GHz (a) for different incidence angles: (**a**) θ = 0°, (**b**) θ = 10°, (**c**) θ = 20°, (**d**) θ = 30°, (**e**) θ = 40°, (**f**) θ = 50°, (**g**) θ = 60°, and (**h**) θ = 70°.

Figure 8 shows the bistatic radar cross section (RCS) measurement results of the copper plate and absorber prototype at 9.26 GHz. The measured bistatic RCS under different incidence angles (*θ* = 0°, 10°, 20°, 30°, 40°, 50°, 60°, 70°) are plotted in Fig. 8. It is observed that both copper plate and metamaterial absorber are specular reflective.

From Fig. 8, absorptivity of incidence angle (θ_i_) can be calculated by

8 $$A({\theta }_{i})\,=\,(1\,-\,\frac{{P}_{t,A}({\theta }_{i})}{{P}_{t,C}({\theta }_{i})})\,\times \,100\quad \quad \quad [ \% ]$$

where P_t,C_(θ_i_) and P_t,A_(θ_i_) are the total relative power reflected from the copper and absorber, respectively.

P_t,C_(θ_i_) and P_t,A_(θ_i_) are estimated from

9 $${P}_{t,C}({\theta }_{i})\,=\,\sum _{{\theta }_{r}=-90^\circ }^{{\theta }_{r}=+90^\circ }{P}_{C}({\theta }_{r})\,=\,{P}_{C}(-90^\circ )\,+\,{P}_{C}(-80^\circ )\,+\,\cdots {P}_{C}(+80^\circ )\,+\,{P}_{C}(+90^\circ )$$

10 $${P}_{t,A}({\theta }_{i})\,=\,\sum _{{\theta }_{r}=-90^\circ }^{{\theta }_{r}=+90^\circ }{P}_{A}({\theta }_{r})\,=\,{P}_{A}(-90^\circ )\,+\,{P}_{A}(-80^\circ )\,+\,\cdots \,{P}_{A}(+80^\circ )\,+\,{P}_{A}(+90^\circ )$$

where P_C_(θ_r_) and P_A_(θ_r_) are the measured relative power at the reflection angle of θ_r_ from the copper and absorber, respectively. P_C_(θ_r_) and P_A_(θ_r_) can be obtained from Fig. 8.

## Discussion

In this work, we proposed an MM absorber using a novel vertically and horizontally symmetric ECS design. Because of the novel ECS design, the proposed absorber is insensitive to incidence angles of both TE and TM polarization. To provide an experimental demonstration, the MM absorber was fabricated on an FR-4 substrate consisting of 20 × 20 unit cells. Under normal incidence, the absorptivity at the specular angle of the proposed MM absorber exceeded 98% at 9.26 GHz even for polarization angles (*ϕ*) in the range 0° to 90°. Under oblique incidence of the TE mode, the absorptivity at the specular angle exceeded 91% at 9.26 GHz even for incident angles (*θ*) varying from 0° to 70°. Under oblique incidence of the TM mode, the absorptivity at the specular angle exceeded 95% despite the incident angle varying from 0° to 70°. The full-wave simulation and measurement thus successfully demonstrated the polarization and angular insensitivities of the proposed MM absorber.

In Table 1, we calculated absorptivity from the measured relative power reflected from the copper and proposed absorber. The proposed MM absorber achieves higher than 90% absorptivity for incident angles up to 70°.

**Table 1:** Calculated absorptivity from the measured relative power reflected from the copper and proposed absorber.

*θ*_i_	0°	10°	20°	30°	40°	50°	60°	70°
P_t,C_(θ_i_)	2.532	2.016	1.502	1.462	1.783	1.816	1.703	3.952
P_t,A_(θ_i_)	0.138	0.102	0.093	0.069	0.175	0.162	0.132	0.449
A(θ_i_) [%]	94.55	94.94	93.81	95.28	90.19	91.08	92.25	88.64

In Table 2, we also compared the absorptivity at the specular angle of the proposed metamaterial absorber with the absorptivity at the specular angle of other angle-insensitive metamaterial absorbers. In particular, the proposed MM absorber achieves higher than 90% absorptivity at the specular angle for incident angles up to 70° for both TE and TM polarization.

**Table 2:** Compared performance at the specular angle of the proposed metamaterial absorber with other angle-insensitive metamaterial absorbers.

Ref	Frequency [GHz]	A at *θ* = 0°		A at *θ* = 50°		A at *θ* = 60°		A at *θ* = 70°		Polarization Insensitivity
		TE	TM	TE	TM	TE	TM	TE	TM	
[27]	10.44	98	98	95	97	90	92	75	89	Yes
[28]	10.91	97	97	N/A	N/A	89	95	N/A	N/A	Yes
[29]	11.3	99	99	95	97	90	99	72	98	Yes
[30]	10.28	98	98	85	97	72	95	N/A	N/A	Yes
[31]	10.14	93	93	97	98	90	97	N/A	N/A	Yes
Proposed Work	9.26	98	98	99	99	97	95	92	95	Yes

## Methods

### Simulation

In order to simulate the performance of the proposed absorber, we used a finite-element method (FEM)-based ANSYS high-frequency structure simulator (HFSS). The proposed metamaterial absorber is a periodic structure. In order to set a unit cell with an infinite periodic array, we assigned two “Master” and “Slave” pairs on the surfaces as boundary conditions in the ANSYS HFSS setup. In addition, we used a Floquet port to excite EM energy to the unit cell, and we calculated the absorptivity from the S-parameters. Because the polarization angle (*ϕ*) and incident angle (*θ*) are defined as “phi” and “theta” on the Floquet port, we simulated the absorptivity for each *ϕ* and *θ* by varying “phi” and “theta” on the Floquet port. We also plotted the magnitudes of the electric fields and vector electric current densities using ANSYS HFSS.

### Measurement

We measured the absorptivity of the fabricated metamaterial absorber in free space, as illustrated in Fig. 6a and b. We calculated the absorptivity from the S-parameters that were measured using an Anritsu MS2038C VNA. We used a single horn antenna to measure S_11_ under normal incidence, as shown in Fig. 6a. We used two horn antennas to measure S_21_ under oblique incidence, as shown in Fig. 6b. The absorber prototype was located 1 m away from the horn antennas in order to satisfy the far-field condition. In addition, the absorber prototype was surrounded by wedge-tapered absorbing materials to remove unwanted reflected and scattered EM waves. In order to receive the reflected EM wave only from the absorber prototype, we applied a time-gating function of the VNA. Before measuring the S-parameters of the absorber prototype, we first measured the S-parameter of the copper plate and set the magnitude of its reflection coefficient to be 1 for the calibration process. In order to measure the absorptivity at different polarization angles, we rotated the horn antenna from 0° to 90°, while fixing its location at *θ* = 0°, and we measured the S-parameters at each polarization angle. In order to measure the absorptivity at different incident angles, the transmitting horn antenna was rotated from 10° to 70° on the azimuth plane. At each incident angle of *θ*, we placed the receiving horn antenna from −90° to 90° to measured relative power reflected from the copper and proposed absorber then we calculated the absorptivity of incidence angle (θ_i_) from Eq. 8.
